# The Emerging Role of PCSK9 in the Pathogenesis of Alzheimer’s Disease: A Possible Target for the Disease Treatment

**DOI:** 10.3390/ijms252413637

**Published:** 2024-12-20

**Authors:** Gabriella Testa, Serena Giannelli, Erica Staurenghi, Rebecca Cecci, Lucrezia Floro, Paola Gamba, Barbara Sottero, Gabriella Leonarduzzi

**Affiliations:** 1 Department of Clinical and Biological Sciences, University of Turin, San Luigi Hospital, 10043 Orbassano, Italy; gabriella.testa@unito.it (G.T.); serena.giannelli@unito.it (S.G.); erica.staurenghi@unito.it (E.S.); rebecca.cecci@unito.it (R.C.); lucrezia.floro@unito.it (L.F.); paola.gamba@unito.it (P.G.); gabriella.leonarduzzi@unito.it (G.L.); 2 Division of Neurology Vand Neuropathology, Fondazione IRCCS Istituto Neurologico Carlo Besta, 20133 Milan, Italy

**Keywords:** Alzheimer’s disease, PCSK9, PCSK9 targeting therapies, LDL receptors, cholesterol, β-amyloid, ApoE, neuroinflammation, oxidative stress

## Abstract

Alzheimer’s disease (AD) is a multifactorial neurodegenerative disease mainly caused by β-amyloid (Aβ) accumulation in the brain. Among the several factors that may concur to AD development, elevated cholesterol levels and brain cholesterol dyshomeostasis have been recognized to play a relevant role. Proprotein convertase subtilisin/kexin type 9 (PCSK9) is a protein primarily known to regulate plasma low-density lipoproteins (LDLs) rich in cholesterol and to be one of the main causes of familial hypercholesterolemia. In addition to that, PCSK9 is also recognized to carry out diverse important activities in the brain, including control of neuronal differentiation, apoptosis, and, importantly, LDL receptors functionality. Moreover, PCSK9 appeared to be directly involved in some of the principal processes responsible for AD development, such as inflammation, oxidative stress, and Aβ deposition. On these bases, PCSK9 management might represent a promising approach for AD treatment. The purpose of this review is to elucidate the role of PCSK9, whether or not cholesterol-related, in AD pathogenesis and to give an updated overview of the most innovative therapeutic strategies developed so far to counteract the pleiotropic activities of both humoral and brain PCSK9, focusing in particular on their potentiality for AD management.

## 1. Introduction

Alzheimer’s disease (AD) is a neurodegenerative disorder that emerged as the most common form of dementia worldwide that chiefly affects over 65-year-old people, although its relevance, the understanding of its etiology, and the development of effective treatments to stop or delay its progression are still few. In accordance with the β-amyloid (Aβ) theory, AD starts with the accumulation in the brain of extracellular Aβ deposits that subsequently leads to tau protein hyperphosphorylation, resulting in tau neurofibril aggregation or neurofibrillary tangles (NFTs) inside neurons and finally in neurodegeneration and both neuronal and synaptic loss [1]. However, Aβ-targeting drugs have been demonstrated to be inadequate, strongly suggesting that other factors concur to AD [2]. Among them, a large body of evidence supports the close association between hypercholesterolemia and AD development, as recently proposed by the “lipid invasion model”, although the mechanisms underlying this link are unclear [3].

The proprotein convertase subtilisin/kexin type 9 (PCSK9) is a protease recognized as one of the major regulators of cholesterol homeostasis by mediating the degradation of hepatic low-density lipoprotein (LDL) receptors (LDLRs), by this way controlling circulating LDL cholesterol (LDL-C) levels. Of note, it was firstly identified in the brain. For these features, PCSK9 could be implied in the cholesterol metabolism disturbances observed in the AD context and would point to its managing as a novel clinical approach potentially helpful for AD cure [4].

The purpose of this review is to present the current knowledge on PCSK9 role in cholesterol dysmetabolism, both in the extra- and intracerebral compartments, and on its impact on AD onset. We also aim at giving a comprehensive overview of the therapeutic strategies presently available to counteract PCSK9 activity; in particular, we discuss their application in the AD treatment and evaluate their possible capability to specifically affect PCSK9 functions inside the brain.

## 2. AD and Cholesterol Homeostasis

Proper cholesterol homeostasis in the brain is essential for brain development and functioning, being this lipid fundamental for the energy supply, membrane turnover and repair, as well as myelination and synaptic transmission. To ensure its optimal amount in the brain, the cerebral cholesterol metabolism is independent from that of peripheral tissues; indeed, plasma and brain cholesterol pools are separated by the blood-brain barrier (BBB) and the blood-cerebrospinal fluid (CSF) barrier, and most of the cholesterol is produced in situ. In adults, astrocytes are the major responsible for cholesterol synthesis, while neurons contribute to a lesser extent. Cholesterol combines with ApoE to form lipoproteins secreted in the extracellular space through the ATP-binding cassette (ABC) transporters, present in the astrocyte membranes, and then delivered to neurons. Among the ABC transporters, ABCA1 is the major one enrolled in the ApoE lipidation and in controlling its steady-state level. In a second step, cholesterol is transferred via ABCG1 to form the so-called high-density lipoprotein (HDL)-like particles. These ApoE-containing lipoproteins are then taken up by LDLRs and low-density lipoprotein receptor-related protein 1 (LRP1), mainly expressed in astrocytes and neurons, respectively, which mediate their supply to neurons and other brain cells. The ApoE-containing lipoproteins bind also with the very low-density lipoprotein receptors (VLDLRs) and by ApoE receptor 2 (ApoER2), but these receptors act primarily as signaling mediators [5]. To keep the brain cholesterol steady-state level, strictly controlled mechanisms exist: excess cholesterol is oxidized into the more hydrophilic oxysterol 24-hydroxycholesterol (24-OHC) that can diffuse across the BBB into the blood stream to reach the liver for further degradation into bile acids. To a lesser extent, brain cholesterol can be also oxidized into another oxysterol, namely 27-hydroxycholesterol (27-OHC). However, most of the brain 27-OHC derives from the circulation by influx through the BBB. The ratio 27-OHC:24-OHC is tightly regulated and, in physiological conditions, it remains constant in the different brain areas. In addition, cholesterol can be complexed with ApoA1-containing lipoproteins and released directly into the CSF via ABC transporters, especially ABCA1 [6].

In pathological conditions, such as AD, several features of cerebral cholesterol metabolism appear altered, bringing about a defective cholesterol content in the brain. Both deficiency and accumulation of cholesterol are detrimental for the correct brain cell activities, as pointed out in cell and animal models of AD as well as in AD patients. On the one hand, it has been reported that brain cholesterol synthesis and amount significantly decreased in *ApoE* knock-out mice, an AD-simulating model characterized by synaptic loss and cognitive dysfunction [7]. Aβ seems to inhibit cholesterol synthesis in primary rat neurons, although cholesterol accumulation co-occurred due to imbalanced intraneural cholesterol trafficking [8], whereas neuronal cholesterol depletion was observed in rat hippocampus and led to changes in Aβ peptide metabolism, excess tau phosphorylation, neuronal oxidative stress, and ultimately neurodegeneration [9]. Conversely, in other investigations, cholesterol loading was observed in senile plaques and cerebral cortices of AD patients [10,11], as well as in the brain mitochondria of AD mouse models, in association with oxidative stress, neuroinflammation, and neuronal damage [12]. Interestingly, the presence of lipoid deposits within neuronal and glial cells, amyloid plaques, and NFTs was already recognized by Alzheimer himself in its first case report of AD [13]. Of note, cholesterol seems to favor amyloidogenesis in the neuronal membrane lipid raft microdomains, where the main proteins involved in Aβ processing are localized [14,15,16], and deletion of astrocyte cholesterol synthesis notably reduced the amyloid burden in neurons of AD mice [17]. In turn, Aβ increases the free cholesterol content in synaptosome lipid rafts and thereby synapse degeneration by promoting cholesterol release from cholesterol ester stores [18]. Moreover, by altering the plasma membrane structure and protein clustering, cholesterol may induce tau amyloid fibrils, as demonstrated by spectrometric characterization of cholesterol-enriched membrane models [19]. The seeming conflict between all these data further underline how complex the impact of the cholesterol network on AD etiology could be.

It has been established that hypercholesterolemia and brain cholesterol dyshomeostasis enhance the risk of AD development. In support of this is mainly the evidence of the very high frequency in late-onset AD subjects of the ε4 allele of apolipoprotein E (ApoE4), an apolipoprotein primarily involved in the lipid transport within the body [20]. *ApoE4* carriers compared to non-carriers are at a higher risk of developing cerebral amyloid angiopathy [21], and of progression from mild cognitive impairment (MCI) to AD [22]. In addition to that, metabolomics, transcriptomic [23], and meta-analysis studies [24] sustained the association between AD and abnormalities in cholesterol metabolism. This evidence suggests that the presence of ApoE4 isoform likely underlies many of the neurodegenerative processes ascribed to cholesterol impairment, including abnormal Aβ deposition, limited Aβ clearance in the CSF, extensive brain atrophy, and reduced dendritic spine density in the hippocampus, all events observed in subjects affected by AD. ApoE4 mouse models exhibited Aβ and hyperphosphorylated tau accumulation, reduced dendritic spine density in the entorhinal cortex, deficits in synaptic transmission, and reduction in Aβ clearance in the CSF [6]. Moreover, cholesterol secretion and trafficking are imbalanced in ApoE4 human astrocytes, despite enhanced cholesterol synthesis [25,26]. Importantly, ApoE appeared to directly bind Aβ and, together with its receptors LDLR and LRP1, to play a key role in astrocyte-mediated Aβ clearance [27,28]. Of note, ApoE4-immortalized mouse astrocytes exhibited lower cholesterol levels than ApoE3 ones [29].

In addition, altered brain cholesterol levels can derive from an uncontrolled exchange of cholesterol between the peripheral circulation and the brain consequent to the BBB damage that takes place in AD patients [30], particularly in *ApoE4* carriers in which AD pathology strongly correlates with cerebral amyloid angiopathy [31]. In this connection, a new theory has been recently postulated to explain AD etiology, the so-called “lipid invasion model”. According to it, AD is the consequence of external lipid entrance into the brain, which in turn derives from insults to the cerebrovascular wall caused by several factors, among which are inflammation, oxidative stress, elevated serum lipid levels, and excessive Aβ-containing lipoproteins rich in cholesterol. Typical features of BBB disruption are reduced junctional strength between blood vessel endothelial cells (ECs), altered expression and activity of metalloproteases and basement membrane proteins, and loss of number and functionality of pericytes [3]. This hypothesis is sustained by some experimental evidence: for example, the hypocholesterolemic agent simvastatin attenuated BBB damage in New Zealand rabbits fed a cholesterol-enriched diet and in rat brain microvascular ECs [32]. Similarly, high cholesterol diet increased BBB permeability in LDLR^−/−^ mice, which were more prone to cognitive impairment than wild-type animals [33], and hyperlipidemic mice overexpressing human amyloid precursor protein (APP) showed impaired Aβ clearance associated to derangements in the cerebral vasculature and BBB [34]. Aβ itself concurs to BBB loosening by disrupting endothelial tight junctions [35], triggering uncontrolled cerebral angiogenesis [36], and upregulating and activating metalloproteases in choroid plexus epithelial cells with consequent leakages in the blood-CSF barrier [37]. In this connection, in rodent models, Aβ deposits observed within the vessels and in the perivascular space, together with loss of pericyte functions, could be responsible for limited blood flow and hypoxia [38]. Moreover, impaired Aβ clearance by LRP1 of the brain microvessel ECs could rise Aβ brain levels, confirming the role of a defective cerebrovasculature in the initiation and progression of AD [39].

## 3. PCSK9 Feature and Pleiotropic Activities

PCSK9 is a serine protease of the subtilase family. In mice and rats, it is mainly expressed in the liver, but also in the intestine, pancreas, kidney, lung, heart, and adipose tissue [40,41,42,43]. In the brain, PCSK9 is highly expressed in mouse embryos, while in adulthood it is only expressed in areas of continuous neurogenesis such as the rostral extension of the olfactory peduncle [44,45]. It is synthesized in the endoplasmic reticulum (ER) as a zymogen of ~72–75 kDa, consisting of an N-terminal pro-domain, a subtilisin-like catalytic domain, and a cysteine- and histidine-rich C-terminal domain, and then autocatalytically cleaved to the 63 kDa mature PCSK9 form still uncovalently bound to its 13 kDa prodomain. The intact heterodimer complex then transits through the Golgi and is finally secreted into the blood stream [46]. At the cell surface, the complex can undergo a further cleavage to a ~50 kDa product by the convertase furin that leads to its inactivation. Therefore, both active and inactive forms of PCSK9 can be present in the plasma, where the active form of PCSK9 is mainly found in association with LDLs, which protect PCSK9 from cleavage by furin [47,48]. It has been suggested that PCSK9 circulates also bound to HDLs and interacts with them, changing their protein, lipid, and glycidic composition, and limiting their cellular cholesterol efflux capacity; moreover, PCSK9 affects HDL antioxidative, anti-apoptotic, anti-thrombotic, and anti-inflammatory activities. However, there is no unanimous consensus on this evidence [49,50,51]. Notably, intracellular cholesterol content is the main factor that controls the expression of the *PCSK9* gene [46].

The role of PCSK9 has been deeply studied in the liver, but there are accumulating findings indicating that it is involved in several molecular and cellular functions in the aforementioned different organs. The members of the proprotein convertase family are responsible for the proteolytic maturation of secreted hormones, cytokines, growth factors, and cell surface receptors; in particular, PCSK9 regulates the expression and/or function of proteins involved in proliferation, apoptosis, inflammation, and cholesterol metabolism. Of note, unlike the other protein convertases, the mature form of PCSK9 remains non-covalently bonded to its inhibitory prosegment, thereby preventing access of other potential substrates to the catalytic pocket. As a result, PCSK9 proteasic activity is impeded and it acts instead as a chaperone to escort specific proteins toward intracellular compartments engaged in degradation [52,53,54,55].

### 3.1. PCSK9 Involvement in Cholesterol Homeostasis

PCSK9 has long been studied due to its role in the transport of circulating LDL and in the regulation of their blood levels since it promotes the degradation of the hepatic LDLRs, which are the major effectors of LDL-C clearance from the blood circulation [56,57]. Because of this, PCSK9 reasonably represents a causal factor in familial hypercholesterolemia [58,59]. The gain of function (GOF) mutations of *PCSK9* are connected with decreased expression of LDLRs on the hepatocyte cell surface and internalization of LDLs that lead to high blood cholesterol levels, while the loss of function (LOF) mutations are associated with increased LDLR levels and internalization of LDLs, resulting in a higher clearance of plasma LDL-C [60]. Of note, the regulation of *PCSK9* hepatic expression appears affected in transgenic and sterol regulatory element-binding protein (*SREBP*)1a, *SREBP*2, and SREBP cleavage-activating protein (*SCAP*) knock-out mice; all these factors are required for the control of cholesterol biosynthesis [61]. The confirmation of a direct role for PCSK9 in lipoprotein metabolism was provided by a series of studies showing that overexpression of *PCSK9* in mice elevated LDL presence in the plasma of control mice but not in that of LDLR-deficient animals [62,63].

In the cell, PCSK9 can act as a chaperone for the transport of the intracellular LDLRs. The LDLR is synthesized in the ER as a precursor of 120 kDa. Passing through the ER and Golgi, the LDLR acquires its mature form undergoing sugar modifications and, if not degraded, it is finally transported to the cell surface. PCSK9 regulates LDLR levels by inducing catalytic degradation of the mature LDLR. The interaction between PCSK9 and the LDLR likely takes place in the ER and allows the trafficking of the PCSK9/LDLR complex to both the post-ER compartments endosomes and Golgi apparatus. By this way, PCSK9 prevents LDLR receptor recycling and conveys it to the lysosome promoting its degradation [42,55,57,58,64].

Extracellularly, secreted PCSK9 interacts with the extracellular domain of the LDLR in the region located in the first epidermal growth factor-like repeat homology domain A (EGF-A), with the aim to post-transcriptionally regulate the LDLR amount inducing its degradation [65]. Indeed, by binding to LDLR, PCSK9 is internalized by the receptor and in turn enhances its intracellular degradation in endo/lysosomal vesicles causing the decrease in cell surface LDLRs [57,66,67].

The ability of PCSK9 to promote LDLR degradation is independent of its catalytic activity, a unique feature among serine proteases. Additional salt bridges are formed between the pro-domain of PCSK9 and the receptor, which acquires an open conformation that prevents it from reacquiring a closed conformation in the endosome; this event precludes the normal recycling of LDLR to the plasma membrane and targets it for lysosomal degradation, resulting in a lower concentration of the receptor on the cell membrane and higher plasma LDL-C levels [65]. In turn, the LDLR regulates PCSK9 trafficking, since the binding of pro-PCSK9 to the precursor form of the LDLR promotes PCSK9 auto-catalytic cleavage [58,68]. The inactive form of PCSK9 has a lower affinity for LDLRs and a reduced capability to degrade them [69].

In addition to LDLR, PCSK9 can bind and modulate the degradation of other specific receptors, such as LRP1, VLDLR, and ApoER2, all involved in cholesterol homeostasis; moreover, it can also bind to the scavenger type B receptor CD36 and plays a direct role in ABCA1-mediated cholesterol efflux through a downregulation of ABCA1 gene and protein expression [67,70,71].

### 3.2. PCSK9 Role in AD

PCSK9 was first identified in primary cerebellar neurons and named as neural apoptosis-regulated convertase-1 (NARC-1) due to its role in apoptotic pathways, but its function in the central nervous system (CNS) is still not fully understood, although literature shows PCSK9 involvement in CNS development, neurodifferentiation [44,72], and in cholesterol metabolism, as observed in cultured brain cells [73]. Interestingly, in mouse brain, the expression of *PCSK9* peaks during cortical and cerebellar development, but drastically abates in adulthood, except in areas of persistent neurogenesis such as rostral extension of the olfactory peduncle and cerebellum [44]. Since, under normal conditions, the BBB prevents the diffusion of plasma lipoproteins and circulating PCSK9 into the brain [72,74], only cerebral PCSK9, which is dynamically regulated, may directly modulate cholesterol homeostasis in the developing brain. However, it cannot be excluded its upregulation in adult individuals in response to AD, stroke, neurovascular diseases, and alcohol-induced neuropathy, events in which protein amounts appear to increase [4,75].

**Figure 1:**
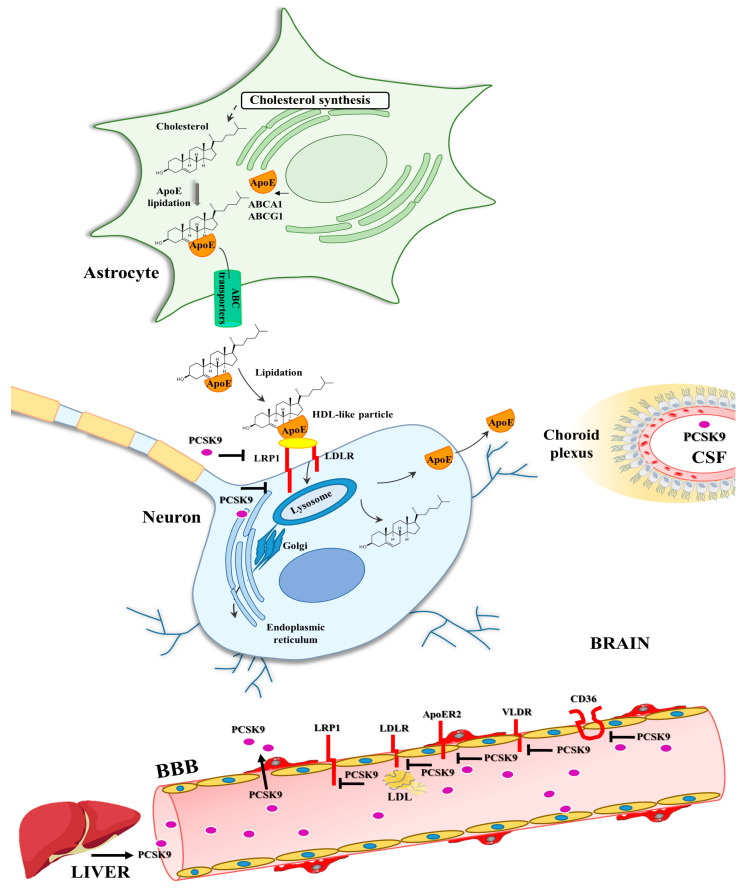
PCSK9 involvement in brain cholesterol dyshomeostasis. Circulating PCSK9 prevents recycling of LDL receptors, inducing hypercholesterolemia, inflammation, and oxidative stress, thus leading to BBB damage that allows PCSK9 to enter into the brain. Inside the brain, PCSK9 affects receptors and transporters involved in astrocyte-synthetized cholesterol and cholesterol uptake by neurons. Abbreviations: ABC, ATP-binding cassette transporter; ApoE, Apolipoprotein E; ApoER2, Apolipoprotein E receptor 2; BBB, blood-brain barrier; CSF, cerebrospinal fluid; HDL, high-density lipoprotein; LDLR, low-density lipoprotein receptor; LRP1, lipoprotein receptor-related protein 1; PCSK9, Proprotein convertase subtilisin/kexin type 9; and VLDLR; very low-density lipoprotein receptor.

PCSK9 interacts with the LDL receptor family members LDLR, LRP1, VLDLR, and ApoER2, which are highly expressed in the brain [67,70,71]. Based on this observation, PCSK9 may be implicated in altered cerebral cholesterol homeostasis due to its degrading activity on the receptors. Moreover, PCSK9 could impact on other steps of the brain cholesterol transport, resulting in a reduced cholesterol uptake by neurons with deleterious consequences for neuronal function and survival [9] (Figure 1); however, an analysis of lipid pathway genes did not reveal any association between PCSK9 and AD progression [76].

Reported evidence about the interplay between these receptors and PCSK9 in an AD context is contradictory. It has been found that overexpression or ablation of *PCSK9* did not modulate the levels of LDLR, VLDLR, and ApoER2 in the hippocampus and cerebral cortex of adult mice [77], while in cultured neurons PCSK9 deficiency reduced LDLR and neurite complexity but did not affect cortical development and behavior in mice [78]. However, LDLR and LRP1 disruption by PCSK9 may be crucial for AD progression since they are the major ApoE receptors in the brain, are involved in lipid homeostasis and intracellular signaling, and are fundamental for the regulation of neuronal structure and synaptogenesis [79]. Indeed, *LDLR*-deficient mice showed hippocampal apoptosis and decreased expression of the presynaptic protein synaptophysin and synaptic integrity in both hippocampal and dentate gyrus, with consequent impairment of spatial cognition [80], reduced hippocampal cell proliferation and synapses formation [81], and signs of impaired learning and memory [82]. In addition, in the brain of *LDLR*-overexpressing transgenic mice, ApoE levels are drastically decreased, improving Aβ clearance [83]. Of note, both *LDLR* and *ApoE* appeared downregulated by PCSK9 during mouse brain development, but not in adulthood, as well as in the lesioned dentate gyrus of adult mice after transient ischemic stroke in association to *PCSK9* overexpression [72]. In humans, a common *LDLR* polymorphism is associated with AD, although only in men [84]. All the aforementioned evidence suggests that PCSK9-mediated pathways could underly the LDLR contribution to brain physiopathological functioning. In confirmation of that, an in vitro investigation pointed out that the inhibition of local LDLR expression by PCSK9 resulted in decreased astrocyte-to-neuron cholesterol transfer with consequent reduction in neuronal cholesterol content and increase in Aβ neurotoxicity [73].

Concerning LRP1, it regulates Aβ efflux through the BBB and in addition its expression in the whole brain and brain capillaries appeared reduced with ageing and in AD, negatively impacting soluble Aβ clearance and leading to amyloid deposition [39,85,86,87]. This could be the consequence of ApoE competition with Aβ for binding LRP1, which might forbid LRP1-dependent metabolism of soluble Aβ [88]. The reduced LRP1-mediated brain-to-blood clearance of monomeric Aβ, due to the receptor degradation by PCSK9, was demonstrated in an established BBB model and further confirmed in an AD mouse model, in prefrontal cortex and hippocampus areas critically involved in memory processing [89]. Since deletion of *LRP1* in mice forebrain neurons led to defective cerebral lipid metabolism, neuroinflammation, synapse loss, and dendritic spine degeneration [90], it is conceivable that similar outcomes could also originate from *LRP1* break-down by PCSK9.

PCSK9 also promotes the degradation of the neuronal receptors VLDLR and ApoER2 [70]. Both receptors are part of the Reelin pathway. By binding with these receptors, processed Reelin fragments modulate microtubule dynamics fundamental for neuronal migration and positioning, and therefore for neocortical lamination, neurite growth, dendritic spine development, and synaptic plasticity of the adult brain. The Reelin pathway was recognized to be involved in AD development since its impairment favors amyloidogenic Aβ processing and tau hyperphosphorylation [91,92]. Moreover, VLDLR contributes to Aβ clearance too; indeed, its gene modulation in astrocytes significantly altered extracellular Aβ content [93].

However, whether PCSK9 contributes to Aβ formation and accumulation or not is still debatable. Indeed, *PCSK9* downregulation in mice enhanced beta-site APP-cleaving enzyme-1 (BACE1) and Aβ levels in the neocortex. PCSK9 has been shown to drive the degradation of non-acetylated BACE1, whose acetylated form constitutes the rate-limiting enzyme in the generation of the toxic Aβ peptides [94,95]. Moreover, in rat PC12 neuron-like cells, a negative correlation has been found between PCSK9 and Aβ levels [96]. In contrast, another study reported no evidence that overexpression or deletion of *PCSK9* regulates BACE1 levels and/or APP processing in the mouse brain, suggesting that PCSK9 effect on AD may be tissue-specific or mediated by other biochemical factors [77].

PCSK9 can also affect the expression of extra-hepatic ABCA1 [97], which is implicated in ApoE secretion at the level of astrocytes; the decrease in astrocyte *ABCA1* expression would induce aberrant lipidation of ApoE-containing particles, which implies improper cholesterol supply to neurons. To confirm the link between the two proteins, a strong decrease in ApoE levels has been observed in both the cortex and CSF of *ABCA1* knock-out mice [98].

### 3.3. Other PCSK9 Activities Implicated in Brain Damage

PCSK9 possesses various pleiotropic activities that also could be implicated in AD neurodegeneration as described below.

#### 3.3.1. PCSK9 and Neuroinflammation

PCSK9 may play a role in boosting neuroinflammation. In U373 human astrocytoma cells treated with Aβ fibrils, PCSK9 significantly increased tumor necrosis factor α (TNFα), interleukin 1β (IL-1β), and IL-6 mRNA levels while *PCSK9* ablation in 5XFAD mice, a model of AD, significantly protected against cognitive impairment induced by Aβ accumulation and attenuated neuroinflammation by decreasing microgliosis and astrocyte reactivity. Of note, knocking out *PCSK9* had minimal impact on the levels of brain cholesterol and of its oxysterol metabolites [99]. The inflammatory response might be triggered by activating nuclear factor kappa B (NF-κB), as suggested by NF-κB translocation to the nucleus of human macrophages induced by *PCSK9* upregulation after oxidized LDL exposure [100]. In supporting that, in a rat model of cerebral ischemia PCSK9 inhibition reversed the release of inflammatory cytokines induced by Toll-like receptor 4 (TLR4) and NF-κB activation [101].

The activation of the NOD-like receptor protein 3 (NLRP3) inflammasome, accompanied by LDLR upregulation, has been demonstrated to increase PCSK9 too. In middle cerebral artery occlusion/reperfusion-subjected mice and oxygen glucose deprivation/reoxygenation-subjected hippocampal neurons, used to simulate cerebral ischemia-reperfusion injury in vivo and in vitro, the NLRP3/PCSK9 machinery promoted inflammation and pyroptosis, evoking brain damage and neurological deficits [102]. Inhibition of *PCSK9* expression prevented the production of TNFα, IL-1β, IL-2, and IL-6 modulated by the TLR4/NF-κB/NLRP3 axis in rat hippocampus [103].

Nevertheless, more recently, anti-inflammatory activity has been ascribed to PCSK9, in particular due to the degradation of CD36 [104]. Indeed, CD36 is involved in fibrillar Aβ-mediated microglia activation and in consecutive stimulation of an innate immune response that leads to the secretion of pro-inflammatory molecules [105].

It cannot be excluded that PCSK9 modulates neuroinflammation also by regulating LDLR and ApoE levels, the last one significantly influencing both innate and acquired immunity. In this connection, in murine BV2 microglial cells and human THP-1 monocytes, ApoE and ApoE mimetics reduced the phosphorylation of the signaling intermediates of inflammation signal transducer and activator of transcription 3 (STAT3), Janus-activated kinase 2 (JAK2), and p38 and p44/42 mitogen-activated protein kinases (MAPKs), and the secretion of TNFα and IL-6 induced by lipopolysaccharide after interaction with LDLRs. The ApoE mimetic’s anti-inflammatory effect was blocked by treatment with PCSK9 due to LDLR degradation by the convertase [106].

#### 3.3.2. PCSK9 and Apoptosis

Apoptosis is known to play a key role in AD pathogenesis [107], and PCSK9 seems to contribute to the death of neurons and glia cells. As described before, PCSK9 was first identified in primary cerebellar neurons and named as NARC-1 due to the observed association between *PCSK9* expression and apoptosis in neurons [44]. Subsequently, the PCSK9 pro-apoptotic activity was confirmed in cerebellar granule neurons (CGNs) [108], although the exact mechanisms are still unclear.

It is likely that whether and how PCSK9 could bring about apoptosis depends on the different cell types and environmental conditions. In both potassium-deprived CGNs and staurosporine-induced CGN models of apoptosis, inhibition of *PCSK9* via RNA interference lowered the levels of activated caspase-3, a key player of the apoptotic cascade, and the mechanism appeared dependent on ApoER2 modulation by PCSK9. However, while in the potassium-deprived CGNs a decrease in c-Jun phosphorylation was also observed, pointing out the involvement of the N-terminal kinase (JNK) apoptotic pathway, in the staurosporine-treated cells, c-Jun phosphorylation was not affected, suggesting that PCSK9-dependent apoptosis could also occur through JNK-independent pathways [109]. *PCSK9* inhibition attenuated neuronal apoptosis after middle cerebral artery occlusion injury in hyperlipidemic mice; again, this effect seemed associated with *ApoER2* upregulation [110]. Of note, ApoER2 itself promotes neuronal survival by inactivating the JNK pathway [111]. In *ApoE*^−/−^ mice fed a high-fat diet, neuronal apoptosis at the hippocampal level was induced by PCSK9 upregulation in association with BACE1 overexpression [112]. In particular, oxidized LDLs, which accumulate in the brain as a consequence of hyperlipidemia [3], can promote neuronal apoptosis through PCSK9 engagement, as assed in PC12 cells; the involvement of the Bcl-2/Bax-caspase 9/3 signaling pathway has been reported [96]. The possibility that ApoE/ApoER2 axis regulates mechanisms essential to neuronal survival in the adult brain is sustained by the evidence that ApoER2^−/−^ mice showed an accelerated loss of corticospinal neurons during normal ageing [113].

Indirectly, PCSK9 could induce neuroapoptosis by interfering with the Reelin/ApoER2 and VLDLR pathways. To suggest this, there is the evidence that, in a mouse model of AD, Reelin-secreting Cajal-Retzius neurons, which are involved in learning and memory processes, undergo caspase-dependent apoptosis [114].

In addition to cerebral cell death, PCSK9 might also bring about EC loss by inducing the apoptotic machinery, thus affecting BBB integrity. After exposure to oxidized LDLs, human umbilical vascular ECs underwent apoptotic death, again through activation of the PCSK9/Bcl-2/Bax-caspase 9/3 pathway [115].

In vitro studies have shown that PCSK9 can also promote cell survival. *PCSK9*-silenced human neuroglioma U251 cells exhibited apoptotic features including cell shrinkage, membrane integrity loss, nuclear fragmentation, and chromatin compaction. These events resulted from activation of caspase-3, decrease of X-linked inhibitor of apoptosis (XIAP) and of the phosphorylation of the anti-apoptotic factor Akt, and increase in Bax/Bcl2 ratio that results in massive cytocrome c release from mitocondria [116].

#### 3.3.3. PCSK9 and Oxidative Stress

The mechanisms through which PCSK9 contributes to oxidative stress in the brain, another typical feature of AD pathogenesis, are not yet clear; however, it has been observed that PCSK9 may exacerbate oxidative stress indirectly by triggering an inflammatory response.

In the brain, PCSK9 overexpression may trigger microglia and astrocyte reactivity, leading to reactive oxygen species (ROS) generation [117]. It has been demonstrated in rats that increased PCSK9 protein levels in the hippocampus contribute to amplify brain oxidative stress and Aβ aggregation, which was accompanied by microglial hyperactivation as well as by BBB breakdown [118].

Moreover, pro-oxidative events might be elicited by PCSK9 thanks to its interaction with CD36, which is expressed on microglia cells and on vascular ECs in the brain of AD patients where it promotes fibrillar Aβ-mediated H_2_O_2_ production [119].

### 3.4. PCSK9 Genotype and AD

Investigations that considered the impact of *PCSK9* genetic variants on neurocognitive impairment and AD led to inconclusive evidence. On the one hand, a DNA analysis in blood, brain tissues, or blood lymphocytes from subjects included in the Quebec Founder Population cohort reported no association of *PCSK9* LOF mutations with AD prevalence or onset [120]. This finding is consistent with the results of a study on Japanese AD patients [121] and with the observations from the PROSPER and REGARDS trials on the elderly population, which both found no correlation between *PCSK9* variants and cognitive performance [122]. In addition, an analysis based on the Mendelian randomization approach in the Danish population enrolled in two different prospective studies evidenced no causal effects of *PCSK9* genetic variants on the risk of AD and also of other forms of dementia and Parkinson’s disease [123]. However, a subsequent genotyping in a multi-cohort study that included also participants of the Quebec Founder Population showed that predisposition to AD was modulated by some *PCSK9* gene variants, albeit in females only [124].

### 3.5. PCSK9 Content in Biological Specimens

In the last decades, several studies measured circulating PCSK9 levels both in animal models and in patients, but the majority of them were conducted in supporting the association between the convertase and cardiovascular diseases [125] or, more generally, its adverse action on the vascular wall, including cerebrovasculature [126]. More limited is the literature specifically concerning PCSK9 protein quantification in the context of AD.

Only one investigation reported that increased serum PCSK9 concentrations reflected Aβ load in the brain of subjects with MCI and AD, pointing to PCSK9 application as a biomarker for AD diagnosis [127]. PCSK9 protein levels were also measured in brain or CSF samples, whose content likely gives more indication of the protein involvement in neurological disorders. Interestingly, in healthy subjects, PCSK9 concentrations in CSF did not significantly correlate with those in plasma and, contrary to the latest ones, did not display diurnal variations, suggesting that cerebrospinal PCSK9 is insensitive to those factors that can affect its amount in peripheral circulation [128]. It has also been reported that AD patients have higher PCSK9 expression and protein levels in the frontal cortex and CSF compared with non-AD subjects, more remarkably in *ApoE4* carriers than in non-carriers, reaching statistical significance in the AD group. In addition, the enzyme amounts positively correlate with ApoE4 levels, suggesting a causative relationship between ApoE4 and PCSK9 [129]. In agreement with this, a very recent investigation showed that in the CSF of AD *ApoE4* carriers, PCSK9 levels were higher compared to non-carriers and a positive correlation was also observed between CSF and serum PCSK9 levels, suggesting PCSK9 exchange between brain and periphery [130]. Examination of normal and late-onset AD autopsied brain samples revealed the upregulation of PCSK9 expression and synthesis in the frontal cortices of patients. In the same investigation, in the CSF of cognitively normal “at-risk” subjects with a parental history of late-onset AD, PCSK9 levels appeared to positively and significantly correlate with ApoE, total tau, and phosphorylated tau levels, but not with those of Aβ_42_ [124]. Neuronal PCSK9 is overexpressed in adult brains during AD, but also in ischemic stroke and neuropsychiatric disorders [131].

In contrast, another investigation found no differences between PCSK9 concentrations in CSF of AD and non-AD individuals; however, PCSK9 levels increased both in AD and non-AD subjects affected by other neurodegenerative processes, and also correlated with total tau levels, pointing to a general PCSK9 involvement in various neurodegenerative diseases [132].

## 4. PCSK9 Targeting Therapies

Currently, the anti-PCSK9 therapies approved for clinical application or still under investigation aim at decreasing systemic cholesterol level by targeting the liver [133]. Two approaches are followed: one is addressed to prevent the interaction between secreted PCSK9 and hepatocyte LDLRs and includes monoclonal antibodies (mAbs), mimetic peptides, and adnectins; the second one, which comprises small interfering RNAs (siRNAs), allele-specific antisense oligonucleotides (ASOs), and clustered regularly interspaced short palindromic repeats (CRISPR) genome editing techniques, is directed to reduce PCSK9 expression, synthesis, or functioning directly within the hepatocytes [4,134,135,136]. Recent progresses in biological therapies further prompted investigations on alternative anti-PCSK9 strategies, among which are macrocyclic peptides, heparin sulfate mimetic peptides, and anticalin fusion proteins [137,138].

### 4.1. Monoclonal Antibodies

Evolocumab and Alirocumab are presently the only two fully human PCSK9-targeting mAbs approved by the US Food and Drug Administration (FDA) and the European Medicines Agency (EMA) for the clinical treatment of hypercholesterolemia thanks to their potency, tolerability, and low immunogenicity. Subcutaneous delivery of them, alone or in combination with other lipid-lowering therapies, drastically reduces plasma LDL-C in dyslipidemic subjects [139,140,141,142]. A third mAb, the humanized semi-synthetic Bococizumab, was suspended due to its higher immunogenicity with time that results in less efficacy with long-term use [143]. All these mAbs bind to PCSK9, and sterically hindering the interaction with the EGF-A domain of LDLR, inhibit its degradation and improve hepatocyte capability to clear blood LDLs [144]. In addition, another injectable mAb, LY 3015014, is under clinical trial; its epitope, unlike the other mAbs, does not include the furin cleavage site in PCSK9, thus allowing its proteolytic degradation. The results of this administration are reduced plasma PCSK9 accumulation. LY 3015014 is characterized by a low clearance that prolongs its action and lowers its dose [145,146].

As an alternative to PCSK9 antibodies, anticalin fusion proteins derived from plasma lipocalins have been created as low-molecular-weight antibody-like drugs able to interfere with the PCSK9 binding to LDLR [147].

### 4.2. Peptide-Based Inhibitors

This modality of drug delivery comprises three classes of inhibitors: (i) nature-derived peptides, which originate from human or plants sources; (ii) combinatorially derived peptides, constructed through phage, mRNA, or other display technologies; (iii) peptidomimetics, whose structure is computationally designed and composition is primarily non-proteinogenic. Nature- and combinatorially derived peptides were the first ones explored as alternative to mAbs for PCSK9 managing [148]. Short sequences of amino acids have been designed to mimic the EGF-A domain of LDLR, thereby annulling its interaction with PCSK9. Some of them are also able to block PCSK9 interaction with VLDLR and less effectively with ApoER2 [149,150,151]. Truncated peptides derived from human PCSK9-prodomain sequences significantly enhanced LDLR levels in vitro without altering PCSK9 content [152]. Enzyme-digested fragments of the legume lupin protein impaired PCSK9-LDLR interaction in hepatocellular carcinoma HepG2 cells [153]. Other mimetic peptides similar to annexin-2, an extra-hepatic endogenous PCSK9 inhibitor, have been developed to hinder the PCSK9 conformational changes necessary for LDLR binding [154]. Cyclic peptides have been synthesized to improve the inhibitory activity toward PCSK9 [148]. Among them, MK-0616 is a macrocyclic peptide that inserts into the LDLR-binding site of PCSK9 and is able to cover a larger portion of it. It has already passed Phase 2 trials and may represent a powerful option for the oral administration of lipid-lowering medication [155,156]. As regards peptidomimetics, N-methyl tetraimidazole derivatives have been also synthesized; they resemble the β-sheet motif of PCSK9 responsible for the contact with the EGF-A domain of LDLR. Among these compounds, RIm13 shows the highest activity in hepatocytes and may be suitable for oral delivery [157,158].

Nevertheless, despite the potentiality of the aforementioned peptide-based inhibitors for PCSK9-targeting interventions, no conclusive validation of their clinical application has been achieved so far, mainly because most of them have a limited oral bioavailability.

### 4.3. Adnectins

Adnectins, also known as monobodies for their smaller size compared to mAbs, are a new class of therapeutics derived from fibronectin; these compounds have been screened for subcutaneous or intravenous delivery. By changing amino acids in their β-sheet loops, they become able to overlap the EGF-A domain binding region of PCSK9 with high affinity and specificity. Among them, Lerodalcibep is a fusion protein of adnectin coupled with albumin to increase its half-life [135,159,160].

### 4.4. Small Interfering RNAs and microRNAs

Unlike the drugs aimed at inhibiting the activity of extracellular PCSK9, siRNAs reduce PCSK9 amount within cells by acting directly on its translation. Indeed, siRNAs are synthetic double-strand RNA molecules targeting selective degradation of protein precursor mRNAs, ensuring a more long-lasting efficacy than protein activity inhibitors. Inclisiran is the first siRNA approved by both US FDA and EMA for the treatment of hypercholesterolemia; the subcutaneous injection of a lipid nanoparticle formulation of the drug, which specifically binds hepatocyte asialoglycoprotein receptors, displays high potency in lowering liver PCSK9 and plasma LDL-C levels [161,162].

MicroRNAs (miRNA) are small, endogenously formed non-coding RNAs modulating several molecular pathways, among which is cholesterol metabolism, and therefore they gained attention for PCSK9 management. Indeed, in HepG2 cells, miR-191, miR-222, and miR-224 have been shown to bind the 3′-end of the *PCSK9* mRNA, lowering its expression; moreover, miR-224 and miR-520d repressed *PCSK9* and increased cell surface expression of LDLRs [163,164]. Of note, miRNA-224-5p inhibits astrocyte neuroinflammatory responses by downregulating NLRP3 inflammasome, thus conferring neuroprotection in AD [165].

### 4.5. Antisense Oligonucleotides

ASOs are short- and single-strand complementary sequences of nucleotides that induce mRNA degradation or pre-mRNA splicing by directly binding to target mRNA, with consequent protein knock-down. Unlike siRNAs, they act primarily in the nucleus. At present, AZD8233 is the only ASO developed for liver PCSK9 management that passed Phase 2 studies, thanks to its safety and potency to lower LDL-C [166]. To further ensure binding affinity and specificity to PCSK9 mRNA and to avoid undesirable side effects, shorter nucleic acid analog ASOs characterized by a locked and thus more stable conformation (locked nucleic acids, LANs) have been considered. Their applicability seems to be possible by evidence in human hepatocytes and non-human primates [167,168].

### 4.6. Small Molecule Inhibitors

Small molecule inhibitors may offer a new promising opportunity to counteract both extra- and intracellular PCSK9, mainly for the advantage of their oral delivery.

A series of chemotypes, characterized by the presence of three amino acid side-chains compatible with LDLR side-chains, have been proposed to prevent the receptor interaction with PCSK9 in hepatocytes [169]; the same interaction appeared to be disrupted by imidazole-based peptide mimetics, among which was Rlm13 [158], and by benzimidazole derivatives [170]. However, all these compounds present some important limitations that have to be overcome to allow their clinical application; for example, they are not selective, and therefore the risk of side effects is major. In addition, considering the fairly flat conformation of the LDLR EGF-A domain, they are not able to firmly insert into its pockets and consequently to ensure high performance [134,135].

Molecules whose principle of action is based upon the regulation of intracellular pathways are under preclinical investigation. A series of benzothiazole small molecules were found to inhibit the *PCSK9* promoter activity in a dose-dependent manner in HepG2 cells [171], while piperidine derivatives have been tested for their ability to inhibit PCSK9 ribosomal synthesis [172]. However, clinical trials of a member of this class, the liver-targeted compound PF-06815345, discontinued after Phase 1 [173]. Alternatively, purine and moracin C derivatives can significantly downregulate *PCSK9* expression [174,175]. More recently, it has been pointed out that S-nitrosylation of PCSK9 inhibits its secretion by hepatocytes into the circulation. Based upon this observation, some molecules have been developed and proven to reduce both serum PCSK9 and cholesterol [176].

### 4.7. Gene Editing

Underway studies are evaluating gene editing and CRISPR technologies to develop optional systems targeting PCSK9. By these platforms it is possible to directly modify the *PCSK9* gene, leading to an LOF genotype which implies a defective PCSK9 activity over a long period of time, possibly even permanently. CRISPR consists in short bacterial DNA sequences followed by fragments of viral nucleic acids that become integrated in the bacterial RNA after genome transcription. CRISPR-RNA complexes with CRISPR-associated (Cas) nucleases, inducing a double-strand break in DNA followed by DNA repair mechanisms and enabling random insertions or deletions that render the gene of interest, in this case PCSK9, permanently dysfunctional. This process, however, is highly inefficient in post-mitotic cells and it can generate unsafe off-target mutations. To improve the efficiency of this approach, CRISPR/base editor tools have been more recently developed; these tools bring precise targeted single-nucleotide changes in the PCSK9 gene without the need for double-strand breaks and DNA repair. In vivo delivery of these CRISPR base editor tools can be also ameliorated by using lipid nanoparticles (LNPs) as carriers, thus avoiding the potential integration of exogenous DNA. In animal and cell models, all these methodologies have given promising results in terms of plasma PCSK9 and cholesterol reduction [177,178,179,180]. In this connection, a clinical trial has been set off in patients with heterozygous familial hypercholesterolemia. The tested VERVE-101 is composed by mRNA encoding an adenine editor encapsulated in LNPs; its administration consists in a single intravenous infusion, and first data are in favor of its safety and efficacy in inactivating the PCSK9 gene and reducing LDL-C [162,181]. Alternatively, in primary mouse hepatocytes, extracellular vesicles (EVs) appeared to be a biocompatible delivery system to vehicle the CRISPR/Cas9 ribonucleoprotein that inactivate the PCSK9 gene [182].

In addition to these tools, virus-mediated gene therapy that employs an adeno-associated virus (AAV), as a gene delivery vector of restriction enzymes, was able to knock down *PCSK9* expression in non-human primates and human hepatocytes, resulting in LDL-C decrease [183].

### 4.8. Vaccines

Active immunotherapy relying on vaccines able to induce high-affinity antibodies that specifically neutralize PCSK9 is also under consideration. This approach can provide lifelong reductions in plasma cholesterol; however, induction of antibody responses against self-antigens, such as PCSK9, might be inactivated by the mechanisms of B cell tolerance and/or might enhance accumulation of auto-reactive T cells. To circumvent that, peptides which mimic regions of the PCSK9 catalytic domain or are involved in its binding with LDLR have been engineered; they have been proven in preclinical studies to develop high titer IgG antibodies against PCSK9 and to decrease plasma LDL-C [184,185,186,187]. Alternatively, negatively charged liposomes have been tested in non-human primates to deliver PCSK9 peptides linked to a tetanus peptide. They resulted in enhancing the humoral response against PCSK9 without provoking systemic inflammation [188].

## 5. PCSK9 Targeting Therapies in AD

The growing evidence demonstrating PCSK9 involvement in AD and the failure of therapies employed so far point to PCSK9 as a target for addressing novel drugs for AD management.

**Figure 2:**
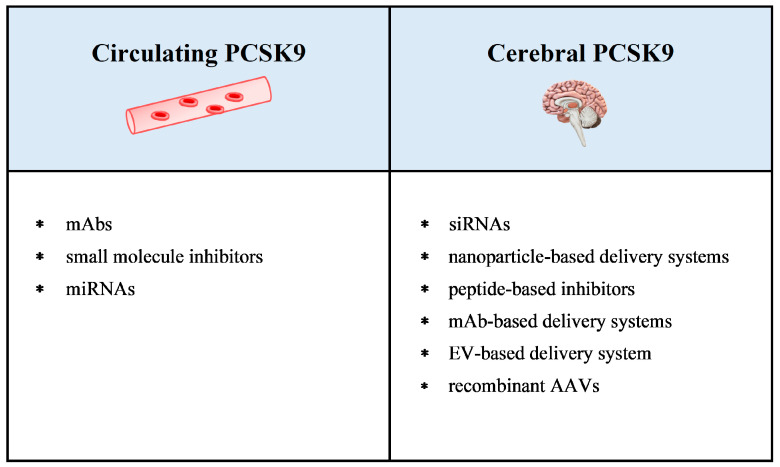
Application of anti-PCSK9 pharmacological tools for AD treatment. Drugs developed to target circulating PCSK9 appear suitable for AD cure by counteracting amyloidogenesis and by reducing hypercholesterolemia, inflammation, and oxidative stress, thus preventing BBB damage. PCSK9-targeting drugs able to cross the BBB could be suggested to delay the neurodegenerative AD progression, exerting their activities directly inside the brain. Abbreviations: AAV, adeno-associated virus; EV, extracellular vesicle; mAb, monoclonal antibody; miRNA, microRNA; and siRNA, small interfering RNA.

Two approaches can be considered (Figure 2). The first one aims at controlling circulating PCSK9 levels and/or its interaction with liver LDL receptors to reduce blood cholesterol and to prevent the hypercholesterolemia-induced BBB damage and the consequent alterations in the brain lipid contents. In addition, this approach could counteract other pleiotropic activities of extra-cerebral PCSK9, such as inflammatory and oxidant responses, that likely contribute to AD development. The second approach aims at controlling intracerebral PCSK9 to maintain brain cholesterol homeostasis, or to limit other PCSK9 activities responsible for neurodegeneration independent of cholesterol metabolism.

### 5.1. Interventions Against Circulating PCSK9 Cholesterol-Related and Non-Related Activities

It is likely that all the hypocholesterolemic pharmacological tools which modulate circulating PCSK9 levels or its functioning are suitable to prevent the effects through which elevated blood cholesterol levels contribute to BBB and brain injuries, limiting AD development. However, only a few of them, namely small inhibitors, mAbs, and miRNAs, have been tested so far in the context of AD, giving promising results in reversing PCSK9 pleiotropic activities known to compromise cerebral physiology.

As proof, subcutaneous injection of the PCSK9 inhibitor SBC-115076 had greater benefit than the cholesterol-reducing drug atorvastatin in improving metabolic disorders of obese rats, because, according to the authors, it could decrease PCSK9 activity in the intestine and cholesterol absorption. The results show an amelioration of BBB breakdown, microglial hyperactivity, hippocampal oxidative stress and synaptic dysfunction, and cognitive decline [118]. The injection of a PCSK9 inhibitor (P-PCSK9, Prep2-8 trifluoroacetate salt) powered down systemic inflammation and ultimately dendritic spine loss via reduction in microglial activation and Aβ aggregation in rats [189].

As regards mAbs, Alirocumab, used to control dyslipidemia, alleviated hippocampal LRP1 expression levels and by this way modulated brain cholesterol homeostasis, reduced hippocampal BACE1 and Aβ42, and restrained cognitive impairment in high cholesterol-fed AD-like rats [190]. Interestingly, either Alirocumab or Evolocumab reverted physiological brain cholesterol homeostasis in hypercholesterolemic patients, as proven by the rise of the ratio between the brain-specific cholesterol metabolites 24-OHC and 27-OHC in the serum. Indeed, this observation is indicative of an increased synthesis of 24-OHC in the brain and of an improved flux of 24-OHC and 27-OHC across the BBB [191]. Recently, the effect of anti-PCSK9 mAbs on changes in plasma levels of LDL-C, small dense LDL (sdLDL-C), and of the oxysterol 7-ketocholesterol (7-KC), a main product of cholesterol autoxidation, has also been investigated; PCSK9 inhibition caused LDL-C and sdLDL-C reduction without significantly affecting 7-KC levels [192]. The evidence may suggest that the drugs, despite their efficacy to lower peripheral cholesterol levels, are not able to act on its oxidation.

The peripheral inhibition of PCSK9 by mAbs reduced, in AD mice, Aβ pathology in the prefrontal cortex and hippocampus areas critically involved in memory processing and prevented disease-related impairment in hippocampus-dependent memory formation, suggesting drug efficacy specifically for AD treatment [89]. Concerning the action on the cells of the blood vessels, administration of Evolocumab to ECs significantly prevented the cytotoxicity induced by H_2_O_2_ [193]. In accordance with this, Evolocumab attenuated vascular ROS generation and improved arterial stiffness in humans [194]. Indirectly, the last study indicates the potential applicability of this PCSK9-targeting drug also in protecting the BBB vascular apparatus from injuries derived by systemic oxidative stress.

Since statins, the most widespread drugs used for LDL-C management, have shown to elicit neurocognitive adverse events, it has been questioned whether also anti-PCSK9 mAbs could perturb cerebral function. Coohort studies carried on to evaluate neurocognition of patients under mAb treatment demonstrated that the incidence of neurocognitive deficits was rare [195]. Conversely, some meta-analyses reported that, though these inhibitors significantly decreased LDL levels, they increased the incidence of neurocognitive impairments, thus suggesting that any impact of PCSK9 activity on the brain was independent from its regulation of peripheral cholesterol [196,197]. In addition to these clinical trials, a long-term, real-world investigation reported no negative impact on the cognitive functions of patients with established atherosclerosis or hypercholesterolemia treated with Alirocumab and Evolocumab. Both mAbs even improved the delayed recall memory, further highlighting the potentiality of PCSK9 management against AD cognitive decline [198]. Overall, additional prospective studies with large samples of individuals are required to determine the long-term safety of these agents, also in consideration that formal procedures for neurocognitive assessments, in terms of study duration, number, and type of assessments during the designated time-period, have not yet been established.

Finally, the upregulation of the *PCSK9*-repressor miR-224-5p appeared to be the mechanism by which the intraperitoneally administration of Diminazene, a modulator of the renin-angiotensin system, inhibited the expression of NLRP3 in astrocytes of AD mice, halting neuroinflammation, alleviating neuronal and synaptic damage, and ameliorating cognitive impairment [165]. In support of the benefits of anti-PCSK9 tools in preserving BBB integrity are the results of a recent investigation where dendritic cell-derived exosomal miR-3064-5p mitigated BBB damage by *PCSK9* inhibition [199].

### 5.2. Therapies Targeting Brain PCSK9 for AD Treatment

It may be questionable whether brain PCSK9-targeting therapies could be meaningful for AD treatment considering that it appeared no longer to be expressed in adulthood [44,45]. According to the database Myeloid Landscape 2 (available online: http://research-pub.gene.com/BrainMyeloidLandscape, accessed on 11 December 2024), the expression of *PCSK9* in various human brain cell types, under different disease conditions, is very low and not significantly different from healthy ones. Nevertheless, the aforementioned data about PCSK9 expression in humans suffering from AD and, more generally, from neurological illnesses (see Section 3.5), suggest an intracerebral production of the protein even in adulthood, at least under pathological conditions [131]. Undoubtedly, knowledge of this aspect is still limited, and further studies are necessary to gain a clearer picture of it. Probably due to these uncertainties, in situ drug administration against intracerebral PCSK9 has not yet been fully explored. To our knowledge, the only example is the stereotaxic injection into the cerebral cortex of hyperlipidemic mice of a lentiviral vector harboring short-hairpin RNA targeting *PCSK9*, which decreased *ApoER2* expression and neuronal apoptosis [110].

Additionally, although healthy BBB impedes PCSK9 entry into the brain [72], it cannot be excluded that several physio-pathological states, such as ageing, infections, cancer, diabetes, atherosclerosis, stroke, and post-traumatic and environmental pollutant-associated injuries [200,201,202,203,204,205], could allow PCSK9 loading into the brain by destabilizing the BBB. In consideration of this, a brain PCSK9-targeting approach would deserve in-depth examination.

For the successful delivery of neurotherapeutic agents, BBB permeability concerns have to be considered [206]. However, many of the drugs developed so far for PCSK9 antagonizing, especially large-size species, are likely unable to reach the brain. For example, mAbs do not typically pass the BBB and indeed, in agreement with this, clinical trials testing their safety did not observe any adverse neurocognitive events [131].

Another critical point is that, to be effective, drug administration to the brain requires a functionally and structurally healthy BBB since its pathological breakdown, which precedes and sustains neurodegeneration, implies perturbations in the endothelial bulk flow transcytosis and in the expression of transporter systems. Consequently, blood-borne medications, including antibodies, proteins, peptides, and small molecules, become trapped within the enlarged perivascular spaces together with other solutes and cannot reach the cerebral cells [30]. For these reasons, though appealing, the use of brain targeting therapies remains a challenge. In this respect, strategies focused on circulating PCSK9 rather than on brain PCSK9 might be more powerful to fight AD since, thanks to their hypocholesterolemic activities, they could prevent BBB disruption, and in the end neurodegeneration.

Hopefully, the coupling with cargo systems such as nanoparticles, cell penetrating peptides, and receptor-targeting peptides, has given promising results in facilitating drug delivery to the brain, at least in in vitro and animal models, since their nanometric size confers them major membrane permeability [206,207]. Nanoparticles comprise a wide series of chemical tools that include quantum dots, dendrimers, metallic nanoparticles, polymeric nanoparticles, carbon nanotubes, liposomes, and micelles. In spite of their different physicochemical features and forms, they share many properties that make them appealing in the clinical practice: high chemical and biological stability, feasibility to be customized according to the chemical nature of the drug, and possibility to be administered via a variety of routes (e.g., oral, intranasal, and parenteral), and to be functionalized to target-specific tissues. Multivalent functionalization with several kinds of ligands, such as antibodies, proteins, or aptamers, improves nanomaterial binding affinity, target specificity, and adaptability to environmental biological stimuli. Of note, nanoparticles functionalized to target brain capillary ECs can favor drug delivery toward the brain without direct BBB crossing. Unfortunately, in vivo trials testing nanomaterial toxicity and immunological compatibility are still limited by the lack of an established evaluation method, thus harmful outcomes cannot be excluded since these materials might accumulate in the liver, kidney, spleen, and brain, eventually causing oxidative damage [206,208].

Peptides also own extreme flexibility for functionalizations that further improve their brain bioavailability by enhancing their lipophilicity and/or lowering their efflux ratio, prolonging their half-life, and improving target specificity [202].

Alternatively, it might be helpful to use a Trojan horse-like strategy in which delivered drugs are linked to mAbs against receptors expressed on the surface of ECs, such as the transferrin receptor, LDLR, and LRP1. Binding the latter, mAbs enable a high specific receptor-mediated transport of drugs which minimize possible side effects [209,210].

Another example of carriers at increasing interest for the treatment of CNS diseases are EVs, among which are small EVs and exosomes. These particles are naturally secreted by most cell types, and therefore are characterized by high biocompatibility and safety profile. Depending on the cell of origin, isolated EVs can be loaded with lipophilic or hydrophilic species, such as proteins, mRNAs, and miRNAs, constituting a nanometric formulation that confers to the agent a high stability in the peripheral circulation and a more selective targeting capability [211].

Recombinant AAVs arguably represent the prominent gene delivery platform for ad hoc CNS clinical applications: they consist of a capsid (outer protein shell) and a cargo (encapsulated genome), both of which can be engineered to obtain better cell type or tissue tropism and control transgene expression, respectively. By these means, transgenes encoding therapeutic proteins, miRNAs, antibodies, and Cas9-guide RNA have been successfully delivered to the brain in humans and animal models where they induced long-term transgene expression. In preclinical studies, intraparenchymal and intracerebroventricular administrations were effective in achieving therapeutic levels of transgene expression in cerebral cells. Intracerebrospinal fluid delivery by intrathecal injection also passes the BBB, but it lacks specificity. Because of the invasiveness of these routes, intravenous and intramuscular administrations were considered, but with lower results and major risks of transduction in peripheral organs. Despite these limits, recent clinical trials demonstrated the relative safety of AAV gene therapy in patients affected by neurological diseases [212,213].

It is conceivable that all these devices might be employed also in the clinical management of intracerebral PCSK9, which is a reason why they should be considered for this purpose, but keeping in mind their applicability also in the case of BBB damage.

## 6. Conclusions

**Figure 3:**
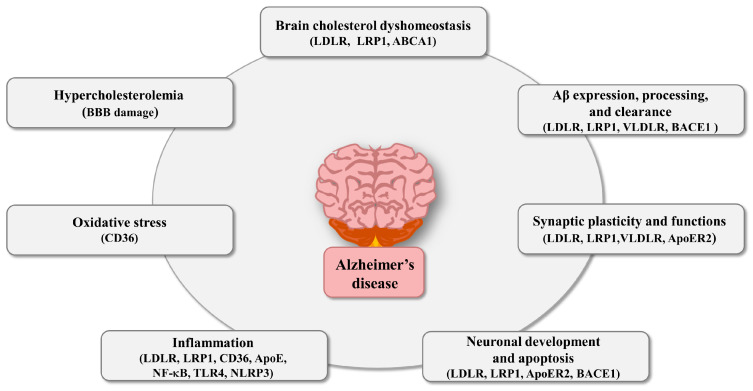
PCSK9 activities associated with Alzheimer’s disease onset and development and the relative factors involved. Abbreviations: ABCA1, ATP-binding cassette transporter A1; ApoE, Apolipoprotein E; ApoER2, Apolipoprotein E receptor 2; BACE1, beta-site amyloid precursor protein-cleaving enzyme-1; BBB, blood-brain barrier; LDLR, low-density lipoprotein receptor; LRP1, lipoprotein receptor-related protein 1; NF-κB, nuclear factor kappa B; NLRP3, NOD-like receptor protein 3; TLR4, Toll-like receptor 4; VLDLR; very low-density lipoprotein receptor.

A large body of literature supports the close association between PCSK9 and AD development. Due to the prominent role played by PCSK9 in the regulation of hepatic LDLR family and blood LDL levels, this protein may represent the link between AD and hypercholesterolemia, an established risk factor for the disease onset. In addition to its involvement in cholesterol metabolism, PCSK9 is also recognized to elicit inflammation and oxidative stress, both events that compromise the BBB integrity and contribute to AD development. Moreover, PCSK9 could promote the neurodegenerative processes by exerting its pro-inflammatory and pro-oxidant activities directly in the brain, where this convertase may be expressed by cerebral cells under pathological conditions [131], and/or accumulate as a consequence of BBB disruption. In addition, PCSK9 is recognized to affect neuronal apoptosis, and to be directly involved in Aβ deposition, by this way furtherly participating in AD development (Figure 3).

For all these reasons, innovative therapies that target circulating and possibly brain PCSK9 may represent a promising strategy for AD clinical management, as indicated by the evidence from in vitro and in vivo studies. In particular, counteracting systemic PCSK9, blood cholesterol levels and inflammatory/oxidative events that contribute to BBB injury could be reduced; for this reason, the systemic PCSK9 targeting could be suggested for AD prevention or treatment during the early prodromal phases, while neutralizing brain PCSK9 could be helpful in delaying full-blown AD progression.

Nevertheless, additional studies are required to determine not only the effectiveness of PCSK9-tergeting therapy in AD cure, but also considering the pleiotropic PCSK9 activities, the long-term safety of its application. Indeed, the occurrence of undesirable side effects has been questioned in terms of cognitive function [196,197] and negative impact on the immune system regulation that could lead to different inflammatory diseases [104].

In addition, the typology of carrier systems that assist drug vehiculation influences both therapy outcomes and hazards. Since the utilization of these tools is recent, careful testing of their toxicity and immunological compatibility is still mandatory before they can be approved for clinical practice.

In conclusion, advances in understanding PCSK9 pitfalls in AD neurodegeneration, integrated with an in-depth characterization of pharmacological tools suitable for the management of this protein, are needed to fight this disabling disorder.

## Abbreviations

7-KC: 7-ketocholesterol; 24-OHC: 24-hydroxycholesterol; 27-OHC: 27-hydroxycholesterol; AAV: adeno-associated virus; ABC: ATP-binding cassette; Aβ: β-amyloid; AD: Alzheimer’s disease; ApoE4: ε4 allele of apolipoprotein E; ApoER2: ApoE receptor 2; APP: amyloid precursor protein; ASO: antisense oligonucleotide; BACE1: beta-site amyloid precursor protein-cleaving enzyme-1; BBB: blood-brain barrier; Cas: CRISPR associated protein; CGN: cerebellar granule neuron; CNS: central nervous system; CRISPR: clustered regularly interspaced short palindromic repeats; CSF: cerebrospinal fluid; EC: endothelial cell; EGF-A: epidermal growth factor-like repeat homology domain A; EMA, European Medicines Agency; ER: endoplasmic reticulum; EV: extracellular vesicle; FDA: Food and Drug Administration; GOF: gain of function; HDL: high-density lipoprotein; IL: interleukin; JNK: c-Jun N terminal kinase; LDL, low-density lipoprotein; LDL-C: LDL cholesterol; LDLR: LDL receptor; LNP: lipid nanoparticle; LOF: loss of function; LRP: LDL receptor-related protein 1; mAb: monoclonal antibody; MCI: mild cognitive impairment; miRNA: microRNA; NARC-1: neural apoptosis-regulated convertase-1; NF-κB: nuclear factor kappa B; NFT: neurofibrillary tangle; NLRP3: NOD-like receptor protein 3; PCSK9: subtilisin/kexin type 9; ROS: reactive oxygen species; sdLDL: small dense LDL; siRNA: small interfering RNA; TLR4: Toll-like receptor 4; TNFα: tumor necrosis factor α; VLDLR: very low-density lipoprotein receptor.
